# A molecular characterization and clinical relevance of microglia-like cells derived from patients with panic disorder

**DOI:** 10.1038/s41398-023-02342-4

**Published:** 2023-02-07

**Authors:** Min-Jung You, Chan Rim, Minji Bang, Soyoung Sung, Hui-Ju Kim, Sang-Hyuk Lee, Min-Soo Kwon

**Affiliations:** 1 Department of Pharmacology, Research Institute for Basic Medical Science, School of Medicine, CHA University, CHA BIO COMPLEX, 335 Pangyo, Bundang-Gu, Seongnam-si, Gyeonggi-do 13488 Republic of Korea; 2 Department of Psychiatry, CHA Bundang Medical Center, CHA University, Seongnam-si, Gyeonggi-do 13497 Republic of Korea

**Keywords:** Molecular neuroscience, Pathogenesis

## Abstract

Few studies report the microglia involvement in the pathogenesis of panic disorder (PD), although the crucial role of microglia in other neuropsychiatric diseases is being emphasized. In addition, there is no report to characterize the phenotypic and functional levels of PD patient-derived microglia to find their clinical relevance. Herein, we used a model to induce patient-derived microglia-like cells (iMGs) to clarify the molecular characteristics and function of PD-iMGs. We established iMGs from 17 PD patients and 16 healthy controls (non-psychiatric controls, HC). PD-iMGs showed increased T-cell death-associated gene-8 expression per the proposal of a previous in vivo study. In addition, we found that patient-derived iMGs showed reduced phagocytosis and increased *TREM2* expression. We analyzed the phenotype of the PD-iMGs by RNA sequencing. The PD-iMGs clustered together distinct from HC-iMGs. Gene set enrichment analysis revealed the involvement of cholesterol biosynthesis and steroid metabolism in PD-iMGs. Regarding the cholesterol synthesis pathway, we discovered *ACAT2* and *DHCR7* as the most impacted genes related to a character of PD-iMGs compared to HC-iMGs. The *ACAT2*, a major cholesterol esterifier, was increased in PD-iMGs. Nevertheless, PD-iMGs did not show lipid droplet accumulation. Interestingly, *ACAT2* expression was inversely correlated with the severity of depression and anxiety sensitivity to publicly observable anxiety reactions. We propose that microglia of PD patients have unique characteristics with dysregulation of cholesterol biosynthesis pathway and impaired phagocytosis, reflecting clinical phenotype.

## Introduction

Panic disorder (PD) is characterized by recurrent, spontaneous panic attacks that occur unexpectedly with incapacitating anxiety and physical sensations of intense fear (e.g., palpitations, chest discomfort, shortness of breath, light-headedness, or fear of losing control). The National Comorbidity Survey-Replication study reported that the lifetime prevalence of panic attacks was 28.3%, and that of PD was approximately 4.7% [1]. As one of the most prevalent psychiatric disorders, PD often follows a chronic course and results in a substantial healthcare burden and individual suffering, particularly when complicated by other psychiatric comorbidities such as agoraphobia and depression [2]. However, the treatment of PD is quite challenging because the neurobiology underlying its pathogenesis is still poorly understood.

Recent evidence suggests that neuroimmune system is closely involved in the development of psychiatric symptoms, ranging from anxiety to psychosis [3]. Microglia are the primary phagocytes within the central nervous system (CNS) parenchyma that centrally mediate neuroinflammatory processes [4]. The evolving concept of microglial biology and its role in modulating neuroimmune have supported that microglia play a crucial role in the pathogenesis of psychiatric disorders [5, 6]. Positron emission tomography imaging studies demonstrated the involvement of neuroinflammation in psychiatric disorders, including psychosis, depression, substance use, and obsessive-compulsive disorder, by showing elevated translocator protein (TSPO) binding in activated microglia [7]. Postmortem brain pathology studies also showed the long-lasting priming and sensitization of microglia in the brains of patients with psychiatric disorders [8]. Particularly, in patients with PD, the association with TSPO polymorphism was reported in a Japanese population [9].

Given that microglia are the major effectors of TSPO-mediated neuroplasticity [10], genetic and functional variations in microglia may influence the pathophysiology of PD. An animal study using mice demonstrated that T-cell death-associated gene-8 (TDAG8), which encodes an acid-sensing receptor highly expressed in microglia, was directly related to the detection and translation of hypercapnia into fear-associated responses [11]. In addition, the level of TDAG8 expression in peripheral blood mononuclear cells (PBMCs) was higher in patients with PD than in healthy individuals and positively correlated with the severity of PD symptoms (according to PD symptom severity scale [PDSS] scores) [12].

However, despite the accumulating evidence, there is a lack of studies investigating the molecular mechanism of the human microglia involvement in PD because of the practical infeasibility of assessing the function of in situ microglia in the brains of living patients. Furthermore, no studies have evaluated the genetic and molecular characteristics of microglia in patients with PD. To address this issue, we established microglia-like cells induced from monocytes obtained from patients with PD. The microglia-like cells (iMGs) have a distinct advantage in that they mirror the intrinsic characteristics of human microglia located in the CNS and reflect a pathological state of ongoing neuroinflammation [13–15]. Although iMGs are not simply assumed to be identical to microglia originating from yolk sac macrophages, transcriptome profiling revealed that iMGs were closely clustered with human primary microglia and those derived from induced pluripotent stem cells [16]. Therefore, it is suggested that iMGs could serve as a potential cellular model system to investigate human microglial pathology in living patients with brain disorders involving microglia.

Here, we evaluated the functional and genetic properties of microglia using the iMGs model in patients with PD compared to healthy controls (non-psychotic control, HC). First, we analyzed acid-sensing TDAG8 expression, phagocytic activity, and the transcriptome of PD-iMGs and their associations with the manifestation of PD symptoms. And then, by clarifying the characterization of PD-iMG cells using transcriptome analysis, we tried to propose additional possible mechanisms involved in TDAG8 in microglia.

## Materials and methods

### Ethics statement

This study was reviewed and approved by the Institutional Review Board of CHA Bundang Medical Center, in accordance with the latest version of the Declaration of Helsinki and the principles of Good Clinical Practice (IRB No. 2019-05-030). All participants provided written informed consent after the study procedures had been fully explained to them.

### Participants

Patients with PD were recruited from the Department of Psychiatry at CHA Bundang Medical Center (Seongnam, Republic of Korea) from August 2019 to May 2021. The diagnosis of PD was made by experienced psychiatrists based on the Diagnostic and Statistical Manual of Mental Disorders, Fifth Edition (DSM-5) criteria [17] using the Structured Clinical Interview for DSM-5 Disorders [18]. Only participants who were diagnosed with primary PD were included. We excluded participants with (1) histories of major psychiatric disorders, including psychotic disorder, substance use disorder, bipolar disorder, and major depressive disorder; (2) histories of neurological disorders, traumatic brain injuries, and intellectual disability (IQ < 70); (3) clinically significant medical illness; and (4) pregnancy. We excluded participants with clinically significant medical illnesses, including cardiovascular, respiratory, endocrine, or immunological diseases. Participants with significant leukocytosis (white blood cell ≧ 10,000/µl) or any signs and symptoms suggestive of acute inflammation, including infection and injury, were also excluded to ensure they were not under inflammatory conditions. HCs were recruited from the local population using online and print advertisements. Individual interviews by trained psychiatrists confirmed that they had no personal or first-degree relative history of psychiatric disorders. The exclusion criteria in this group were the same as those used for patients with PD. All participants were of Korean descent. 17 patients with PD and 16 HCs were finally included in this study.

The severity of state and trait symptoms in patients with PD was measured at baseline. State symptoms were assessed using the PDSS [19], Beck Depression Inventory-II [20], and Beck Anxiety Inventory [21]. Symptoms associated with PD trait vulnerability were assessed using the Anxiety Sensitivity Index-Revised (ASI-R) [22], the harm avoidance (HA) subscale from the Temperament and Character Inventory [23], and the neuroticism subscale from the Neuroticism-Extraversion-Openness Personality Inventory [24]. The characteristics of the study participants are presented in Supplement Table 1.

**Table 1:** Statistics of correlation analysis among the clinical indicators, gene expression levels, and phagocytic function.

		TDAG8	TREM2	ACAT2	DHCR7	GRN	Phagocytosed beads
PDSS	Correlation Coeefficient	−0.396	−0.766*	−0.359	0.088	−0.231	−0.097
	Sig. (two-tailed)	0.202	**0.013**	0.228	0.786	0.447	0.792
	N	12	**10**	12	12	11	10
BDI-II	Correlation Coeefficient	−0.186	−0.657*	−0.732**	−0.392	−0.003	0.267
	Sig. (two-tailed)	0.564	**0.044**	**0.008**	0.207	0.993	0.488
	N	12	**10**	**12**	12	11	10
BAI	Correlation Coeefficient	−0.539	−0.444	−0.205	0.06	−0.041	0.133
	Sig. (two-tailed)	0.07	0.199	0.545	0.854	0.894	0.732
	N	12	10	12	12	11	10
ASI-R-FRS	Correlation Coeefficient	−0.692*	−0.442	−0.091	0.259	0.137	−0.55
	Sig. (two-tailed)	**0.015**	0.2	0.79	0.417	0.655	0.125
	N	**12**	10	12	12	11	10
ASI-R-FPOAR	Correlation Coeefficient	−0.099	−0.343	−0.702*	−0.296	−0.055	−0.311
	Sig. (two-tailed)	0.76	0.333	**0.016**	0.351	0.858	0.415
	N	12	10	**12**	12	11	10
ASI-R-FCS	Correlation Coeefficient	−0.021	−0.086	−0.357	−0.345	−0.133	−0.167
	Sig. (two-tailed)	0.948	0.814	0.281	0.272	0.664	0.667
	N	12	10	12	12	11	10
ASI-R_FCD	Correlation Coeefficient	−0.277	−0.541	−0.583	−0.156	−0.122	0.227
	Sig. (two-tailed)	0.384	0.107	0.06	0.628	0.69	0.557
	N	12	10	12	12	11	10
HA	Correlation Coeefficient	0.25	−0.183	−0.425	−0.165	−0.617*	0.633
	Sig. (two-tailed)	0.434	0.613	0.193	0.608	**0.025**	0.067
	N	12	10	12	12	**11**	10
Neuroticism	Correlation Coeefficient	0.23	−0.037	−0.33	−0.163	−0.613*	0.407
	Sig. (two-tailed)	0.472	0.919	0.321	0.613	**0.026**	0.277
	N	12	10	12	12	**11**	10

*PDSS* Panic Disorder Severity Scale, *BDI-II* Beck Depression Inventory-II, *BAI* Beck Anxiety Inventory, *ASI-R* Anxiety Sensitivity Index-Revised, *FRS* fear of respiratory symptoms, *FPOAR* fear of publicly observable anxiety reactions, *FCS* fear of cardiovascular symptoms, *FCD* fear of cognitive dyscontrol, *HA* harm avoidance, *TDAG8* T cell death-associated gene 8, *TREM2* Triggering receptor expressed on myeloid cells 2, *ACAT2* Acetyl-CoA C-acetyltransferase 2, *DHCR7* 7-Dehydrocholesterol reductase, *GRN* Granulin precursor.

### Establishment of microglia-like cells

Briefly, peripheral blood (~30 mL) was collected using a heparinized tube from HCs and PD patients. PBMCs were isolated using Lymphoprep^TM^ (STEMCELL technologies, Catalog# 07851/07861) density gradient centrifugation and were resuspended in Roswell Park Memorial Institute (RPMI) 1640 buffer (Gibco, Waltham, MA, USA) containing 10% FBS (Gibco) and 1% penicillin /streptomycin (Gibco). The isolated PBMCs were seeded at a density of 5 × 10^5^ cells/ml for overnight in incubator with 5% CO_2_ at 37 °C. The remaining PBMCs were used for qPCR analysis. And then, the media was carefully aspirated, and adherent cells (monocytes) were cultured in RPMI 1640 Glutamax (Gibco) supplemented with 1% penicillin/streptomycin (Gibco), recombinant human granulocyte-macrophage colony-stimulating factor (GM-CSF; 10 ng/ml; R&D Systems, Minneapolis, MN), and recombinant human interleukin-34 (IL-34; 100 ng/ml; R&D Systems) for 21 days to establish iMGs based on several published protocols [13, 14, 25, 26]. Each person-derived iMGs showed different cell yields despite of high expression of microglia signature genes. Thus, the extent of molecular analysis was determined depending on the obtained individual iMGs yield and quality control (QC) (Supplementary table 1). The validation of iMGs was verified by ramified morphology, the increased mRNA expression of microglial signature genes (*Mafb, Csf1r, Gpr34, Hexb, C1qa*) compared to individual monocyte, and immunofluorescence study for microglia marker (P2RY12).

### Quantitative polymerase chain reaction (qPCR)

Total RNA was extracted using TRIzol reagent (Invitrogen, Catalog# 15596018) and evaluated using a NanoDrop spectrophotometer (DeNovix, Catalog# DS-11FX). cDNA was synthesized using a RevertAid First Strand cDNA Synthesis Kit (Thermo Scientific™, Catalog# K1622). To assess the microglial signature in iMGs and PBMCs, we analyzed the expression of *MAFB*(F: 5′-TCAACGACTTCGACCTGCTC-3′, R: 5′-GTGTCTTCTGTTCGGTCGGG-3′), *CSF1R*(F:5′-ATTCATCAACGGCTCTGGCA-3′, R: 5′-AGGACCTCAGGGTATGGGTC-3′), *C1QA*(F: 5′-TCCCGGGAATTAAAGGCACC-3′, R: 5′-ACCGTGTCGAAGATGACCAC-3′), *GPR34*(F” 5′-CCGCCACAAAACTTCTCAGC-3′, R: 5′-CCAACCAGTCCCACGATGAA-3′), and *HEXB*(F: 5′-GATGTTGGCGCTGCTGACTC-3′, R: 5′-GGGCTGTGGCTGATGTAGAA-3′) and *GAPDH* (PPH00150F) genes. Also, we analyzed the expression of *TREM2* (PPH06065E), *TDAG8* (PPH12137A), *ACAT2* (Bioneer, Korea, S-6042-S200-ACAT2), *DHCR7* (S-6042-S200-DHCR7), and *GRN* (*GRN-F*: CCTGGACCCCGGAGGAGC and *GRN-R*: ACGGTAAAGATGCAGGAGTGG). cDNA was amplified using Power SYBR Green PCR Master Mix with primers using Applied QuantStudio^TM^ Design & Analysis Software v1.5.1 (Thermo Fisher Scientific) at 95 °C for 10 min, followed by 40 cycles of 15 s at 95 °C and 1 min at 60 °C. A melting curve was generated to examine the specificity of the amplification. The relative quantity (RQ) levels were calculated with the 2^−ΔΔ Ct^ method using glyceraldehyde 3-phosphate dehydrogenase as the standard internal control.

### Immunocytochemistry

The cells were fixed with 4% paraformaldehyde and were permeabilized with 0.3 % Triton X-100 for 10 min. Indirect immunofluorescence was performed using the following primary antibodies: rabbit anti-IBA1 (Wako; #019-19741; 1:400), rabbit anti- purinergic receptor P2Y12 (P2RY12; Abcam; ab140862; 1:200), rabbit anti-transmembrane protein 119 (TMEM119; Novus; NBP2-30551,1:200), rabbit anti-TDAG8 (Alomone labs; AGR-043; 1:1000). The cells were incubated with the primary antibodies diluted in 0.3% Triton X-100 in PBS containing 10% bovine serum albumin and 3% fetal bovine serum at 4 °C overnight. After rinsing thrice with PBS for 5 min, Alexa 488- or Alexa-594-conjugated secondary antibodies (Abcam) were used for detection. Nuclei were counterstained with 4′6-diamidino-2-phenylindole (DAPI; Sigma). Cells without the addition of primary antibodies served as negative controls. Fluorescent images were taken using a confocal microscope (LSM 700, Carl Zeiss, Jena, Germany).

### 4,4-difluoro-1,3,5,7,8-pentamethyl-4-bora-3a,4a-diaza-s-indacene (BODIPY) staining

To observe lipid droplet accumulation in iMGs, we conducted BODIPY staining and modified Marschallinger’s work [27]. In brief, BV2 cells were seeded on a 5 × 10^4^-cell 24-well plate, and iMGs derived from 2 × 10^5^ PBMCs were analyzed. To generate a positive control for BODIPY staining, following 18-h lipopolysaccharide (LPS, 5 uM; sigma) treatment, BV2 cells were fixed with 4% PFA for 10 min and washed in DPBS. iMGs, LPS-treated BV2 cells, and non-treated BV2 cells were incubated in PBS with BODIPY493/503 (1:1000, Thermofisher) for 10 min at room temperature. Cells were washed in DPBS and mounted on slides with a DAPI mountant. At least three randomly selected fields were photographed (x40 magnification) using confocal microscopy (TCS SP5, Leica).

### Phagocytosis assay

To quantify phagocytosed beads of iMGs, optimally cultured iMGs for 21 days were treated with 2 μl red fluorescent latex beads (2 μm, Sigma-Aldrich, St. Louis, MO, USA) for 2 h at 37 °C. Phagocytic activity was then stopped by adding 2 ml ice-cold PBS. The cells were washed twice with ice-cold PBS, fixed, stained with a microglial marker (IBA-1), and counterstained with DAPI. The cells were analyzed using confocal microscopy (TCS SP5-**II**, Leica).

### Enzyme-linked immunosorbent assay (ELISA)

To quantify the plasma concentration of C-reactive protein, we used a C-reactive protein ELISA kit (R&D systems, #DCRP00) according to the manufacturer’s instructions.

### RNA sequencing and data analysis

10 HC-iMGs and 14 PD-iMGs that passed raw read data quality control (QC) were selected for RNA sequencing. RNA sequencing was analyzed referring to our previous study [28]. Gene classification was based on searches done using the DAVID (http://david.abcc.ncifcrf.gov/) and Medline databases (http://www.ncbi.nlm.nih.gov/). The clustering heatmap was generated using MeV software (https://mev.tm4.org). For the gene set enrichment analysis (GSEA), we used a GSEA program developed by the Broad institute with a molecular signature database [29, 30].

### Statistical analysis

To more precisely describe statistics, we identified statistical outliers and conducted normality testing. Statistical outliers were eliminated using the ROUT test (Q < 0.5%). Cleaned data (with eliminated statistical outliers) sets were tested with the Kolmogorov–Smirnov and Shapiro–Wilk tests to test their normality. The statistical significance of between-group differences was assessed using an unpaired *t* test and a paired t-test to compare microglia-specific genes between PBMCs and iMGs. In addition, we conducted a spearman correlation analysis between panic symptom severity and gene expression using GraphPad Prism version 7 for Mac (GraphPad, La Jolla, CA). Statistically significant differences are indicated as follows: ****P < 0.0001, ***P < 0.001, **P < 0.01, and *P < 0.05.

## Results

### The increased TDAG8 expression in iMGs derived from patients with PD

**Fig. 1:**
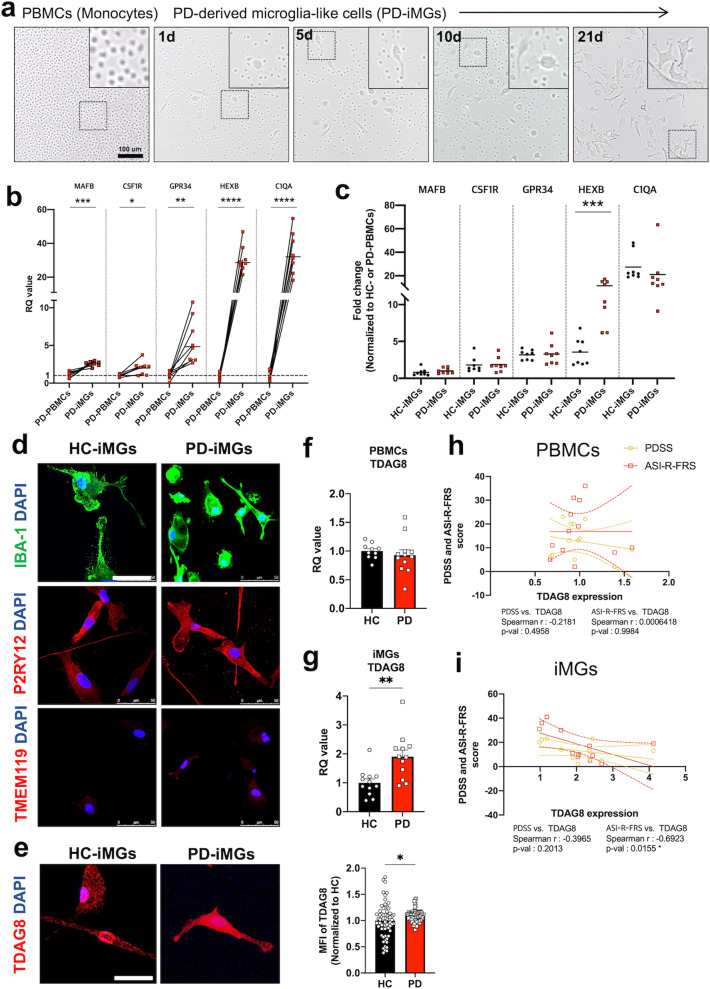
The validation of generating iMGs derived from PD patients and TDAG8 expression. **a** The representative images (×20) of PBMCs and iMGs during differentiation processes. Each day indicated the time after the differentiation factor was added. Scale bar = 100 um. The white boxes represent magnified images. **b** Validation of iMGs compared to PBMCs by qPCR. **c** Comparison of microglia signature genes between HC-iMGs and PD-iMGs. The RQ values are the ratio of respective genes as a percentage of PD-PBMCs. **d**, **e** The representative images (40x) of iMGs stained with microglia-specific markers and TDAG8. The bar graph (right-hand side of **e**) indicates mean fluorescence intensity of TDAG8, Scale bar = 50 um. Each point represented the immunofluorescence intensity of a cell to analyze TDAG8 protein level analyses and (**f**, **g**) qRT-PCR analysis for TDAG8 mRNA expression in PBMCs and iMGs of both HC and PD. Each dot indicates a person-derived iMGs or PBMCs. **h**, **i** Correlation analysis between TDAG8 mRNA expression and clinical indicator of panic symptoms. The data shown are as mean ± standard error of the mean (SEM). To compare statistical significance between the two groups, we conducted a paired *t* test for (**b**), unpaired *t* test for (**c, e, f, g**), and spearman correlation analysis. *P < 0.05, **P < 0.01, ***P < 0.001, and ****P < 0.0001 compared with the PBMCs and HC independently.

Demographic description of included donors was shown in Supplementary Table 1. There is no difference of age and serum C-reactive protein level between two group. To generate iMGs from the peripheral blood of patients with PD and HCs, we isolated PBMCs and cultured them for 21 consecutive days with human IL-34 and GM-CSF, which is essential for microglial survival and differentiation. As shown in Fig. 1a, we observed morphological changes in PMBCs from small and spherical morphology to enlarged and branched morphology over time. Next, we conducted real-time quantitative (qRT)-PCR for microglia-specific genes to confirm iMG generation. Patients with PD showed a greatly increased expression of microglia-specific genes, including *Mafb, Csf1r, Gpr34, Hexb*, and *C1qa* in iMGs compared to PBMCs (Fig. 1b). In addition, there were no significant differences on the increment of these microglia-specific genes between HC-iMGs and PD-iMGs except *Hexb* (Fig. 1c). In the immunofluorescence study, we found the strong expression of P2RY12 and the weak expression of TMEM119 in the iMGs of both groups (Fig. 1d). Based on the previous reports that showed an increased expression of TDAG8 in PBMCs of patients with PD [12], we compared the expression of TDAG8 between patients with PD and HCs in PBMCs and iMGs, respectively. TDAG8 expression did not differ between HC-PMBCs and PD-PBMCs. However, PD-iMGs showed higher TDAG8 expression than HC-iMGs in qPCR and immunofluorescence (Fig. 1e–g). TDAG8 expression in PD-iMGs showed a significant inverse correlation with the fear of respiratory symptoms from the ASI-R, which refers to fundamental fears of anxiety-related sensations [31] (Table 1). There were no significant correlations between the severity of state symptoms and TDAG8 expression in PD-PBMCs and PD-iMGs (Fig. 1h, i).

### Reduced phagocytic function and triggering receptor expressed on myeloid cells 2 (TREM2) mRNA elevation in PD-iMGs

**Fig. 2:**
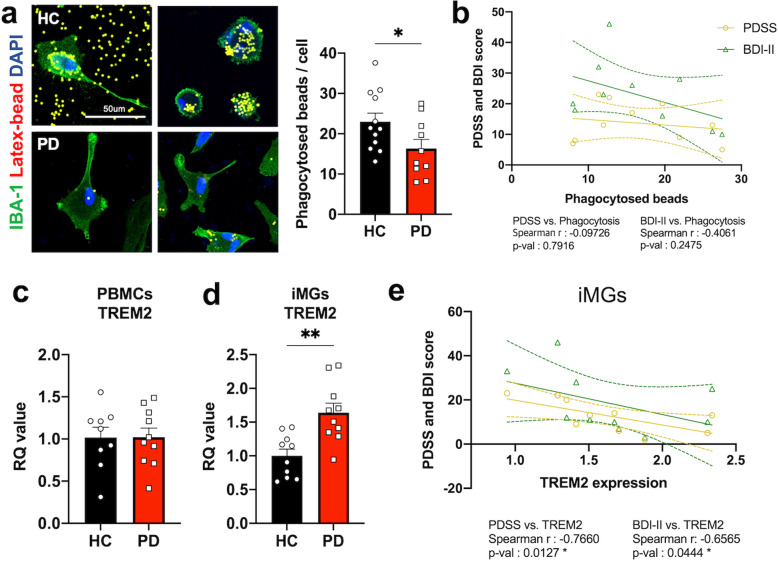
The phagocytic function relevance of PD-iMGs to panic symptoms. **a** The representative images (×40) of HC- and PD-iMGs during phagocytosis. The bar graph (Right side of images) indicates phagocytosed beads in HC and PD respectively (For quantification, 2 fields per subject were used; total subjects HC = 12, PD = 10). **b** Correlation analysis between phagocytosed beads and clinical indicators of panic symptoms. **c**, **d** qRT-PCR analysis for TREM2 expression in PBMCs and iMGs of both HC and PD. **e** Correlation analysis between TREM2 mRNA expression and clinical indicator of panic symptoms. The data shown are as mean ± standard error of the mean (SEM). To compare statistical significance between the two groups, we conducted an unpaired *t* test and spearman correlation analysis. *P < 0.05, and **P < 0.01 compared with the HCs.

We conducted a phagocytosis assay using latex beads to assess the representative microglial and phagocytic function in iMGs. We found that PD-iMGs engulfed the latex beads less than HC-iMGs (Fig. 2a). Phagocytic activity was not directly correlated with the severity of PD-associated state and trait symptoms (Fig. 2b). Considering that TREM2 plays a pivotal role in the phagocytic function of microglia [32], we hypothesized that decreased phagocytic activity of PD-iMGs would be associated with decreased TREM2 expression. Unexpectedly, PD-iMGs showed increased TREM2 expression compared to HC-iMGs; no significant differences were found between PBMCs of patients with PD and HCs (Fig. 2c, d). Interestingly, TREM2 expression in PD-iMGs was negatively correlated with the severity of panic symptoms and comorbid depression (Fig. 2e).

### The transcriptome revealed the dysregulation of the cholesterol biosynthesis pathway in PD-iMGs

**Fig. 3:**
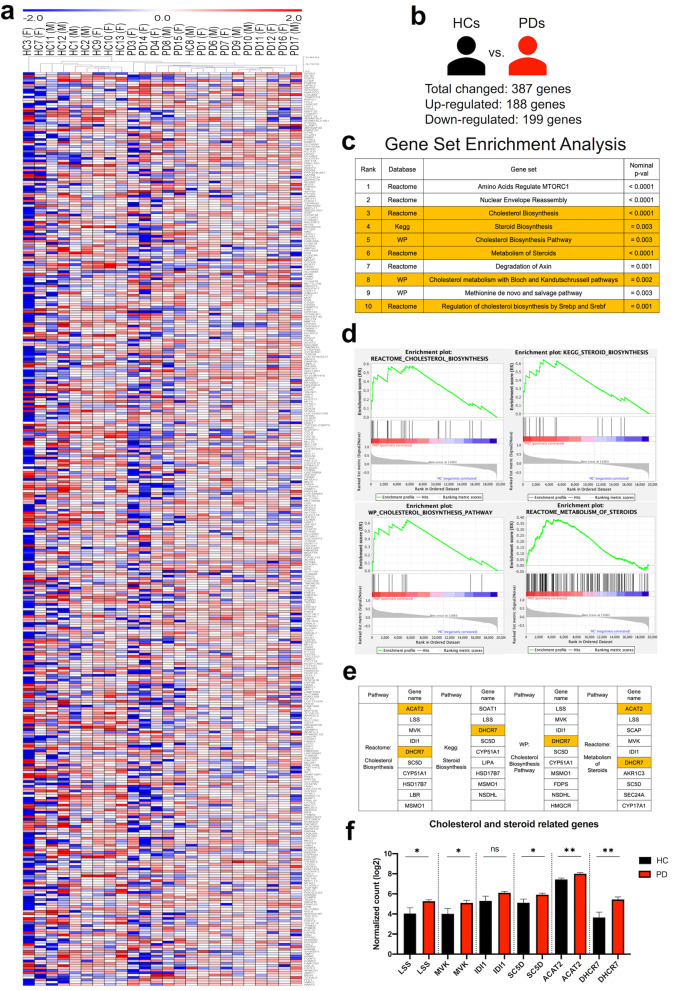
Transcriptome analysis of PD-iMGs. **a** Hierarchical clustering and heatmap of differentially expressed genes between HC- and PD-iMGs. Fold change >1.5, *P*-value <0.05. **b** Graphical index of the number of differentially expressed genes between HC- and PD-iMGs. **c** The top 10 enriched pathways are annotated among databases. Cholesterol and steroid biosynthesis pathways were highlighted in orange color. **d** The top four gene set enrichment plots in GSEA rank. **e** Core genes are enriched in the top four pathways. The overlapped genes among pathways are shown in orange color. **f** Unpaired *t* test of logarithmic normalized read count of overlapped genes between HCs and PDs. To compare statistical significance between the two groups, we conducted an unpaired *t* test. *P < 0.05, and **P < 0.01.

Comparative transcriptome analysis was conducted to elucidate molecular differences in iMGs between patients with PD and HCs. We identified that 387 genes were differentially expressed in PD-iMGs with statistical significance based on the differentially expressed genes (DEGs) with a fold change >1.5 and a P-value <0.05. Among the 387 genes, 199 were downregulated, and 188 were upregulated (Fig. 3b).

Using a hierarchical clustering analysis with DEGs, we found that PD-iMGs were clustered together and distinct from HC-iMGs (Fig. 3a). GSEA revealed a set of biological processes changed and affected by DEGs. We listed the top 10 changed biological processes documented in three databases (Reactome, KEGG, and WikiPathways; Fig. 3c). Six of ten biological processes were involved in cholesterol biosynthesis and steroid metabolism. We selected the four top ranks of the gene set and analyzed the core gene of each set. As shown in Fig. 3d, several DEGs of PD-iMGs were annotated and enriched in the top-ranked cholesterol and steroid biosynthesis pathways. Based on these results, we listed each pathway’s top 10 enriched genes and selected six shared genes including *Lss*, *Mvk*, *Idi1*, *Sc5d*, *Acat2*, and *Dhcr7*. To narrow down the candidate of the most impacted genes to PD-iMGs, we compared the normalized counts of each shared gene between HCs and PDs. The expression of these six genes was upregulated in PD-iMGs except IDI1. Since *ACAT2* and *DHCR7* showed strong statistical significance, we selected them as the most impacted genes related to a character of PD-iMGs compared to HC-iMGs (Fig. 3e, f).

### New possible target: ACAT2 in PD-iMGs

**Fig. 4:**
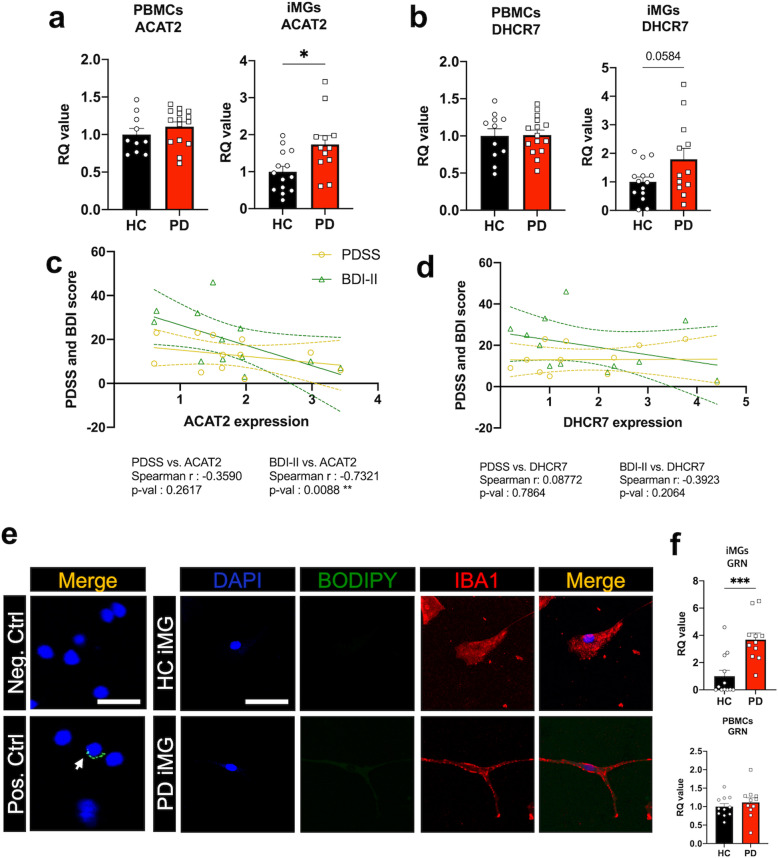
Novel targets based on the transcriptome of PD-iMGs and their clinical relevance. **a**, **b** qRT-PCR analysis for cholesterol biosynthesis genes in PBMCs and iMGs of both HC and PD. **c**, **d** Correlation analysis between ACAT2 and DHCR7 mRNA expression levels and clinical indicators of panic symptoms. **e** The representative images (40x) of BODIPY-stained BV2 cells and iMGs. LPS-treated and non-treated BV2 cells were used for positive and negative controls, respectively. Scale bar = 50 um. **f** qRT-PCR analysis of *GRN* expression in PBMCs and iMGs respectively. To compare statistical significance between the two groups, we conducted an unpaired *t* test and spearman correlation analysis. *P < 0.05, **P < 0.01, and ***P < 0.001.

Based on our transcriptome analysis, RNA sequencing revealed that cholesterol biosynthesis genes, specifically *ACAT2* and *DHCR77*, were upregulated in PD-iMGs. There were no significant changes in *ACAT2* and *DHCR7* expression in PD-PBMCs compared to HC-PBMCs. On the other hand, we found increased *ACAT2* expression in PD-iMGs compared to HC-iMGs (Fig. 4a, b). *DHCR7* did not achieve statistical significance although it showed increasing trend (*p* = 0.0584). *ACAT2* expression was inversely correlated with the severity of depression and anxiety sensitivity to publicly observable anxiety reactions (Fig. 4c, Table 1). On the other hand, *DHCR7* expression showed no relevance with the severity of state symptoms (Fig. 4d, Table 1).

To identify the association between increased expression of *ACAT2 and DHCR7* and the accumulation of lipid droplets in iMGs, we conducted BODIPY staining to quantify the accumulated cholesterol and lipid metabolites in iMGs. As shown in Fig. 4e, PD-iMGs did not show lipid droplet accumulation. To find the possible mechanism underlying this result, we examined the expression of *GRN*, a direct inhibitor linked to lipid droplet accumulation in PD-iMGs [27]. PD-iMGs showed increased *GRN* expression compared to HC-iMGs (Fig. 4f) although there were no significant changes in *GRN* expression in PD-PBMCs compared to HC-PBMCs. Of note, *GRN* expression showed negative correlations with the severity of trait symptoms, including HA and neuroticism (Table 1). Additionally, to analyse the interaction between *Acat2* and candiate genes evoked in PD-iMGs, we used siRNA to knockdown (KD) ACAT2 in BV2 cells (siACAT2). As shown in Supplementary materials (S2), siACAT2 increased *TDAG8* and *TREM2* expression. In addition, siACAT2 showed a higher cAMP generation compared to siCON, suggesting that ACAT2, TREM2, and TDAG8 are at least interwinded, affecting each other.

## Discussion

To our knowledge, this is the first to report the molecular characteristics of microglia-like cells derived from patients with PD. Our finding of altered TDAG8 expression in PD-iMGs is consistent with previous studies [11, 12] and provides further evidence that microglial TDAG8 may mediate panic symptoms by affecting anxiety sensitivity to respiratory symptoms. We found that PD-iMGs exhibited reduced phagocytosis and had unique transcriptome features distinct from HC-iMGs. GSEA revealed that genetic changes in the cholesterol biosynthesis pathway characterize the clustering of PD-iMGs. Notably, the expression level of several genes, including *ACAT2*, was associated with the severity of state and trait symptoms of PD in PD-iMGs.

TDAG8 is one of the potential key markers to explain the involvement of microglia in the pathogenesis of PD. A previous study reported increased TDAG8 expression in PD-PBMCs, which positively correlated with the severity of PD symptoms [12]. Although we found no significant difference in TDAG8 expression between PD-PBMCs and HC-PBMCs, PD-iMGs showed significantly higher TDAG8 expression than HC-iMGs. The discrepancy on no significant difference in TDAG8 expression between PD-PBMCs and HC-PBMCs compared to a previous study [12] might be stemmed from different demographics and smaller number of study group. Previous study was conducted with average age of 27.7, however, average age of 34.4 in our study. Nevertheless, our findings represent a meaningful advance in that iMGs obtained from living patients with PD are better proxy cells reflecting the brain’s physiology than PBMCs. Given that iMGs mirror microglia in the human brain [13, 16], it could be inferred that patients with PD have microglial TDAG8 overexpression.

Furthermore, we found a significant inverse association between the level of TDAG8 expression and the severity of anxiety sensitivity to respiratory symptoms. At first glance, this finding seems contradictory to the previous finding of a positive correlation between TDAG8 expression in PD-PBMCs and PDSS scores [12]. This may be due to the inherent differences between PBMCs and iMGs. However, our results could be explained more plausibly by a compensatory response to deal with excessive sensitivity to respiratory stimuli. Anxiety sensitivity is a personality trait with genetic heritability that confers susceptibility to PD by amplifying fear of anxiety-related sensations [33, 34]. An animal study demonstrated that TDAG8 knock-out mice showed less severe CO_2_-evoked panic-like responses than wild-type mice. This suggests that microglial acid-sensing via TDAG8 could mediate the manifestation of panic symptoms by sensitizing hypercapnia-induced responses and contribute to increasing anxiety sensitivity to respiratory symptoms, consistent with increased TDAG8 expression in PD-iMGs. In the same vein, our finding of an inverse correlation between TDAG8 expression in PD-iMGs and ASI-R scores to respiratory symptoms might be understood as a compensatory effort in patients with PD who have high anxiety sensitivity to attenuate their anxiety reactions.

In addition to TDAG8, we proposed *ACAT2* and *DHCR7* as candidate genetic markers related to PD in microglia. 7-dehydrocholesterol reductase (DHCR7) is an enzyme that synthesizes cholesterol from 7-dehydrodesmosterol, while Acetyl-CoA Acetyltransferase 2 (ACAT2), a major cholesterol esterifier, reduces the excessive cellular level of cholesterol to prevent their toxic effect, leading to an increase of cholesterol esters (CEs). The CEs formed by ACAT2 are involved in the formation of lipid droplets, which can be hydrolyzed by a neutral, cytoplasmic CE hydrolase. Thus, the increase in ACAT2 would serve as a defensive mechanism to convert excessive cholesterol synthesis to CE.

We also found that TREM2 was increased in PD-iMGs. It has been reported that TREM2, a well-known factor related to phagocytosis and synaptic pruning, also regulates excessive cholesterol [35]. Loss of TREM2 causes dysregulated cholesterol transport and metabolism in microglia, leading to pathogenic lipid accumulation [35]. Despite of increase of ACAT2 in PD-iMGs, PD-iMGs did not show lipid-droplet formation. However, progranulin (GRN), a direct negative regulator of lipid droplet formation, was increased in PD-iMGs. Therefore, it might be speculated that both *TREM2* and *GRN* elevation contribute to the suppression of lipid droplet accumulation in PD-iMGs despite increased ACAT2. This relationship is supported by the negative correlations between the TREM2, ACAT2, and GRN expression in PD-iMGs and the severity of state and trait symptoms. Elevated *ACAT2* expression was associated with less severe depression and anxiety sensitivity to publicly observable anxiety reactions, and *GRN* expression was inversely correlated with the severity of trait symptoms, including HA and neuroticism. TREM2 elevation in PD-iMGs showed negative correlations with the severity of PD symptoms and comorbid depression. Although the complex mechanisms underlying these associations cannot be determined here, compensatory reactions to reduce excessive lipid accumulation by genetic faults associated with cholesterol metabolism and enhance phagocytosis to eliminate aberrant synaptic connections underlying the development of PD might be actively occurring in the brain of patients with PD.

Regarding a link between TDAG8 and the cholesterol biosynthesis pathway, there is a report that TDAG8 accelerates cholesterol accumulation in an atherosclerotic mouse model via proliferation and migration in vascular smooth muscle cells [36]. In addition, TDAG8-deficient mice show reduced cholesterol metabolism in T helper cells [37]. Notably, macrophages with mutant TDAG8 showed impaired lipid droplet turnover [38], indicating that TDAG8 plays a critical role in maintaining phagosomal and lysosomal pH in microglia. Notably, we confirmed that siACAT2 induced the increase of TDAG8 and TREM2 mRNA expression in BV2 cell, indicating that these genes are interconnected in microglia. Thus, *ACAT2* elevation might be a compensatory response to normalize *TDAG8* overexpression in PD-iMGs although *ACAT2* elevation seem not to be enough to restore TDAG8 overexpression. However, the further study will be needed which factor increased TDAG8 expression in microglia of PD. Collectively, all of the targets (TDAG8, ACAT2, DHCR7, TREM2, and GRN) presented in this study appear to be directly or indirectly involved in cholesterol biosynthesis and metabolic pathway in PD-iMGs. Also, GRN is a possible suppressor of phagocytosis [27], indicating that *GRN* upregulation might be linked to reduced phagocytosis.

Phagocytosis, as a representative microglial intrinsic function, is required for proper neural circuit formation and maintaining CNS homeostasis [39]. By phagocytizing apoptotic cells and cellular debris, microglia help the CNS to maintain an optimal environment in a healthy state. In our results, PD-iMGs exhibited reduced phagocytosis, which might be involved in the reduced phagocytic elimination of synapse. The disturbance of synaptic pruning is linked to the psychopathology of multiple complex neuropsychiatric disorders, including autism spectrum disorder, schizophrenia, attention deficit hyperactivity disorder, and bipolar disorder [6]. Pharmacotherapy such as benzodiazepines in PD could also lead to the removal of excitatory synapses by microglial phagocytosis of synaptic proteins in vitro and in vivo [40]. Although there is no report on whether PD exhibits a change in synaptic density and synaptic pruning in live or postmortem brain tissue, our results suggest that PD might also be associated with an impaired synaptic network. However, further study will be required to determine whether reduced phagocytosis is a causal pathomechanism of PD because the aberrant phagocytosis might be a visible outcome of the distorted cholesterol pathway in microglia.

Although our study, the first to evaluate the molecular characteristics of PD-iMGs, was restricted to a small discovery cohort, our results strengthen previous studies and propose add-on therapeutic targets in PD. In addition, psychological assessments have not been conducted in HCs since they were ensured to have no significant signs and symptoms of psychiatric disorders by individual interviews at the time of recruitment. However, by clarifying the molecular character of PD-iMGs, our results support that targeting cholesterol biosynthesis pathways in microglia might be another therapeutic strategy in PD. Additionally, a further study needs to be conducted to determine whether the impaired cholesterol metabolism is limited to microglia or is a global phenomenon observed in PD because several studies reported the serum cholesterol or CE alteration in major depressive disorders [41] as well as PD patients [42, 43]. Furthermore, the animal study is required that microglia-specific ACAT2 KD is associated with PD-relevant behaviors via TDAG8 overexpression. Finally, although PD-iMGs did not show lipid droplet accumulation, whether they have lipid droplet accumulation prone character should be investigated.

## Supplementary information

Supplementary table

Supplementary method and result

## Data Availability

RNA-seq datasets have been deposited online in Gene Expression Omnibus (GEO) under accession number GSE212802. Other clinical data were included in supplementary table 1.
