# Supplementary material for: A molecular characterization and clinical relevance of microglia-like cells derived from patients with panic disorder
Source: Transl Psychiatry. 2023 Feb 7;13:48. doi: 10.1038/s41398-023-02342-4 (PMC9905570; doi:10.1038/s41398-023-02342-4)
Supplement: Supplementary file 1 — Supplementary table [file 41398_2023_2342_MOESM1_ESM.docx]

**Supplementary Table 1.** Characteristics of the study participants

| **Participants** | | **Sex**^a^ | **Age**^a^ | **Age of onset** | **Agora-phobia** | **PDSS** | **BDI-II** | **BAI** | **ASI-R-FRS** | **ASI-R-FPOAR** | **ASI-R-FCS** | **ASI-R-FCD** | **HA** | **Neuro-ticism** | **Plasma CRP**  **(ng/ml)** | **Analysis** |
| --- | --- | --- | --- | --- | --- | --- | --- | --- | --- | --- | --- | --- | --- | --- | --- | --- |
| PD1 | | F | 56 | 54 | No | 7 | 20 | 21 | 11 | 19 | 10 | 3 | 18 | 8 | - | iMG: QuantSeq, phagocytic function, qPCR |
| PD2 | | M | 50 | 45 | Yes | 17 | 26 | 46 | 21 | 15 | 8 | 3 | 22 | 11 | 134.3 | iMG: phagocytic function, qPCR |
| PD3 | | F | 41 | 25 | No | 9 | 28 | 18 | 10 | 19 | 10 | 5 | 23 | 6 | 11.22 | iMG: QuantSeq, phagocytic function, qPCR |
| PD4 | | F | 30 | 23 | Yes | 23 | 32 | 45 | 41 | 26 | 19 | 20 | 32 | 11 | - | iMG: QuantSeq, phagocytic function, qPCR |
| PD5 | | M | 30 | 24 | No | 23 | 33 | 30 | 9 | 16 | 5 | 12 | 33 | 11 | 18.56 | iMG: qPCR |
| PD6 | | M | 36 | 13 | Yes | 13 | 25 | 36 | 19 | 16 | 21 | 12 | 31 | 12 | 193.4 | iMG: QuantSeq, qPCR |
| PD7 | | F | 26 | 25 | No | 5 | 10 | 15 | 2 | 6 | 1 | 1 | 28 | 10 | 34.38 | iMG: QuantSeq, phagocytic function, qPCR |
| PD8 | | M | 42 | 37 | No | 13 | 11 | 14 | 17 | 22 | 21 | 4 | 28 | 10 | 73 | iMG: QuantSeq, phagocytic function, qPCR |
| PD9 | | M | 61 | 60 | Yes | 6 | 7 | 7 | 5 | 5 | 0 | 0 | 23 | 6 | 164.9 | iMG: QuantSeq, qPCR |
| PD10 | | M | 16 | 13 | No | 2 | 3 | 4 | 8 | 8 | 1 | 0 | 7 | 2 | 16.85 | iMG: QuantSeq, qPCR |
| PD11 | | F | 50 | 50 | No | 14 | 10 | 36 | 30 | 6 | 8 | 0 | 17 | 9 | 526.7 | iMG: QuantSeq, , qPCR |
| PD12 | | F | 40 | 25 | Yes | 20 | 12 | 40 | 31 | 8 | 8 | 12 | 32 | 10 | 353.9 | iMG: QuantSeq, qPCR |
| PD13 | | M | 25 | 23 | Yes | 20 | 16 | 28 | 24 | 24 | 0 | 10 | 25 | 7 | 562 | iMG: phagocytic function |
| PD14 | | F | 22 | 20 | Yes | 22 | 46 | 49 | 36 | 14 | 4 | 21 | 12 | 9 | 629.1 | iMG: QuantSeq, phagocytic function, qPCR |
| PD15 | | F | 24 | 22 | Yes | 8 | 18 | 0 | 34 | 20 | 14 | 7 | 17 | 5 | 4796 | iMG: QuantSeq, phagocytic function |
| PD16 | | F | 20 | 19 | No | 20 | 37 | 37 | 25 | 21 | 7 | 13 | 29 | 10 | 29.51 | iMG: QuantSeq, phagocytic function, qPCR |
| PD17 | | M | 21 | 17 | Yes | 13 | 23 | 21 | 11 | 19 | 10 | 3 | 18 | 8 | 25.48 | iMG: QuantSeq, qPCR |
| Summary | | M/F: 8:9 | 34.2 ± 13.8 | 29.1 ± 14.5 | Y/N: 9:8 | 13.8 ± 6.8 | 21.0 ± 11.8 | 26.3 ± 15.3 | 19.6 ± 11.8 | 15.5 ± 6.7 | 8.6 ± 6.9 | 7.4 ± 6.7 | 23.2 ± 7.6 | 8.5 ± 2.6 | 496.3 ± 257.8 |  |
| HC1 | | M | 42 |  |  |  |  |  |  |  |  |  |  |  | - | iMG: QuantSeq, phagocytic function, qPCR |
| HC2 | | M | 27 |  |  |  |  |  |  |  |  |  |  |  | 305.8 | iMG: QuantSeq, phagocytic function, qPCR |
| HC3 | | F | 30 |  |  |  |  |  |  |  |  |  |  |  | - | iMG: QuantSeq, phagocytic function, qPCR |
| HC4 | | M | 35 |  |  |  |  |  |  |  |  |  |  |  | 37.67 | iMG: phagocytic function |
| HC5 | | F | 40 |  |  |  |  |  |  |  |  |  |  |  | 67.88 | iMG: phagocytic function, qPCR |
| HC6 | | F | 34 |  |  |  |  |  |  |  |  |  |  |  | 12.98 | iMG: phagocytic function |
| HC7 | | F | 38 |  |  |  |  |  |  |  |  |  |  |  | 1316 | iMG: QuantSeq, qPCR |
| HC8 | | M | 26 |  |  |  |  |  |  |  |  |  |  |  | 34.38 | iMG: QuantSeq, phagocytic function, qPCR |
| HC9 | | F | 28 |  |  |  |  |  |  |  |  |  |  |  | 13.87 | iMG: QuantSeq, phagocytic function, qPCR |
| HC10 | | F | 23 |  |  |  |  |  |  |  |  |  |  |  | 285.2 | iMG: QuantSeq, phagocytic function, qPCR |
| HC11 | | M | 40 |  |  |  |  |  |  |  |  |  |  |  | 490 | iMG: QuantSeq, phagocytic function, qPCR |
| HC12 | | M | 49 |  |  |  |  |  |  |  |  |  |  |  | 134.8 | iMG: QuantSeq, phagocytic function, qPCR |
| HC13 | | F | 46 |  |  |  |  |  |  |  |  |  |  |  | 640.2 | iMG: QuantSeq, phagocytic function, qPCR |
| HC14 | | F | 62 |  |  |  |  |  |  |  |  |  |  |  | 223.5 | iMG: qPCR |
| HC15 | | F | 41 |  |  |  |  |  |  |  |  |  |  |  | 41.07 | iMG: qPCR |
| HC16 | | F | 43 |  |  |  |  |  |  |  |  |  |  |  | 33.98 | iMG: qPCR |
| Summary | | M/F: 6:10 | 37.75 ± 2.5 |  |  |  |  |  |  |  |  |  |  |  | 259.8 ± 96.37 |  |
|  | PD, panic disorder; HC, healthy control; PDSS, Panic Disorder Severity Scale; BDI-II, Beck Depression Inventory-II; BAI, Beck Anxiety Inventory; ASI-R, Anxiety Sensitivity Index-Revised; FRS, fear of respiratory symptoms; FPOAR, fear of publicly observable anxiety reactions; FCS, fear of cardiovascular symptoms; FCD, fear of cognitive dyscontrol; HA, harm avoidance; PBMC, peripheral blood mononuclear cell, iMG, induced microglia-like cell; qPCR, quantitative polymerase chain reaction; ICC, immunocytochemistry.  ^a^ There were no significant differences in sex and age between patients with PD and HCs (sex: *χ*^2^ = 0.4233, *p* = 0.5153; age: Mann-Whitney *U* = 110.5, *p* = 0.367); Chi-square test and Mann-Whitney U test were conducted with R 4.2.0. | | | | | | | | | | | | | | | |
