# Supplementary material for: A molecular characterization and clinical relevance of microglia-like cells derived from patients with panic disorder
Source: Transl Psychiatry. 2023 Feb 7;13:48. doi: 10.1038/s41398-023-02342-4 (PMC9905570; doi:10.1038/s41398-023-02342-4)
Supplement: Supplementary file 2 — Supplementary method and result [file 41398_2023_2342_MOESM2_ESM.docx]

**Supplementary material and method**

**Cell line culture**

Mouse microglial cell line BV2 were culture in Dulbecco’s modified eagle medium (DMEM) supplemented with 10% fetal bovine serum and 1% penicillin/streptomycin. BV2 cells were incubated at 37 °C in a 5% CO2 incubator. The culture medium was changed every 2–4 days and routinely checked no detection of mycobacterium.

**siRNA transfection**

BV2 cells were plated onto 6-well plate with density of 10^6 cells per wells for transfection. We used siGENOME^TM^ siACAT2 (Dharmacon, M-042806-01-0005) for silencing target gene, siGENOME^TM^ non-targeting control (Dharmacon, D-001210-02-05) for negative control, and Lipofectamine RNAiMAX (Invitrogen, 13778075) following manufacturer’s protocol. BV2 cells were cultured with siRNAs for 48 hr followed by qRT-PCR and ELISA. Non-target siRNA transfected BV2 cells referred as siCON BV2, and siACAT2 transfected cells were referred as siACAT2 BV2 cells.

**Introducing acidic environment**

After siACAT2 knockdown, siCON and siACAT2 BV2 cells were incubated with stably maintained pH media established and used in a previous study [1]. In brief, DMEM powder was dissolved without sodium bicarbonate in distilled water and mixed with 10 mM HEPES (4–1-piperazineethane-sulfonic acid) and 10 mM MES (2-ethanesulfonic acid) (Sigma). After titrating to the target pH 6.8 with NaOH or HCl, sodium bicarbonate and 1% penicillin/streptomycin were supplied. Cells were incubated at 37 °C in a 5% CO2 incubator for 30 minutes. These cells referred as siCON+pH6.8 and siACAT2+pH6.8.

**Enzyme linked Immunosorbent assay (ELISA)**

To obtain intracellular cyclic adenosine monophosphate (cAMP), siCON+pH6.8 and siACAT2+pH6.8 cells were harvested with 0.1M HCl. cAMP in the freshly harvested samples quantified using cAMP ELISA kit (Enzo, ADI-900-067A), according to the manufacturer’s instructions.

1. **B.**

**Supplement figure 1. Acat2 knockdown increases Tdag8 expression and induces a higher acidosis-mediated cAMP generation**

(A) qRT-PCR analysis for Acat2, Trem2, Grn, and Tdag8 in siCON and siACAT2 BV2 cells. (B) Quantification of intracellular cAMP concentration in siCON with pH6.8 media and siACAT2 with pH6.8 media. To compare statistical significance between the two groups, we conducted an unpaired t-test. *P<0.05, **P<0.01, ***P<0.001, and ****P<0.0001.

**Reference**

1.Jang, K.-B. *et al.* Persistent Acidic Environment Induces Impaired Phagocytosis via ERK in Microglia. *Neurochem Res* 1–13 (2022) doi:10.1007/s11064-022-03533-z.
